# Lineup fairness: propitious heterogeneity and the diagnostic feature-detection hypothesis

**DOI:** 10.1186/s41235-019-0172-5

**Published:** 2019-06-13

**Authors:** Curt A. Carlson, Alyssa R. Jones, Jane E. Whittington, Robert F. Lockamyeir, Maria A. Carlson, Alex R. Wooten

**Affiliations:** Texas A&M University – Commerce, PO Box 3011, Commerce, TX 75429 USA

**Keywords:** Eyewitness identification, Simultaneous lineup, Lineup fairness, Diagnostic feature-detection hypothesis, Propitious heterogeneity

## Abstract

Researchers have argued that simultaneous lineups should follow the principle of propitious heterogeneity, based on the idea that if the fillers are too similar to the perpetrator even an eyewitness with a good memory could fail to correctly identify him. A similar prediction can be derived from the diagnostic feature-detection (DFD) hypothesis, such that discriminability will decrease if too few features are present that can distinguish between innocent and guilty suspects. Our first experiment tested these predictions by controlling similarity with artificial faces, and our second experiment utilized a more ecologically valid eyewitness identification paradigm. Our results support propitious heterogeneity and the DFD hypothesis by showing that: 1) as the facial features in lineups become increasingly homogenous, empirical discriminability decreases; and 2) lineups with description-matched fillers generally yield higher empirical discriminability than those with suspect-matched fillers.

**Electronic supplementary material:**

The online version of this article (10.1186/s41235-019-0172-5) contains supplementary material, which is available to authorized users.

## Significance

Mistaken eyewitness identification is one of the primary factors involved in wrongful convictions, and the simultaneous lineup is a common procedure for testing eyewitness memory. It is critical to present a fair lineup to an eyewitness, such that the suspect does not stand out from the fillers (known-innocent individuals in the lineup). However, it is also theoretically possible to have a lineup with fillers that are too similar to the suspect, such that even an eyewitness with a good memory for the perpetrator may struggle to identify him. Our first experiment tested undergraduate participants with a series of lineups containing computer-generated faces so that we could control for very high levels of similarity by manipulating the homogeneity of facial features. In support of two theories of eyewitness identification (propitious heterogeneity and diagnostic feature-detection), the overall accuracy of identifications was worst at the highest level of similarity. Our second and final experiment investigated two common methods of creating fair lineups: selecting fillers based on matching the description of the perpetrator provided by eyewitnesses, or matching a suspect who has already been apprehended. A nationwide sample of participants from a wide variety of backgrounds watched a mock crime video and later made a decision for a simultaneous lineup. We found that description-matched lineups produced higher eyewitness identification accuracy than suspect-matched lineups, which could be due in part to the higher similarity between fillers and suspect for suspect-matched lineups. These results have theoretical importance for researchers and also practical importance for the police when constructing lineups.

## Background

Mistaken eyewitness identification (ID) remains the primary contributing factor to the over 350 false convictions revealed by DNA exonerations (Innocence Project, 2019), and is a factor in 29% of the over 2200 exonerations nationally (National Registry of Exonerations, 2018). As a result, psychological scientists continue to study the problem, researching aspects of the crime as well as the ID procedure and other issues. Here, we investigate how police should select fillers for lineups in order to maximize eyewitness accuracy.

A lineup should be constructed so that the suspect does not stand out, with reasonably similar fillers (e.g., Doob & Kirshenbaum, 1973; Lindsay & Wells, 1980; Malpass, 1981; National Institute of Justice, 1999). Often the goal is to reduce bias toward the suspect in a lineup (Lindsay, 1994), but sometimes the issue of too much filler similarity is addressed. For example, Lindsay and Wells (1980) found that using fillers that matched the perpetrator’s description, as opposed to matching the suspect, reduced false IDs more than correct IDs (see also Luus & Wells, 1991). They concluded that eyewitness ID accuracy is best if the fillers do not match the suspect too poorly (see also Lindsay & Pozzulo, 1999) and do not match the suspect too well, as they can when matched to the suspect rather than description of the perpetrator.

This recommendation to avoid a kind of upper limit of filler similarity is based largely on investigating the impact of different filler selection methods (e.g., match to description versus match to suspect) on correct ID rates separately from false ID rates. Usually the recommended procedure is the one that reduces the false ID rate without significantly reducing the correct ID rate (e.g., Lindsay & Pozzulo, 1999). However, Clark (2012) showed that these kinds of “no cost” arguments do not hold under scrutiny. The true pattern of results that arises when manipulating variables to enhance the performance of eyewitnesses is a tradeoff, such that a manipulation (e.g., unbiased lineup instructions, more similar fillers, sequential presentation of lineup members) tends to lower both false and correct IDs.

The best method for determining whether system variable manipulations are producing a tradeoff or actually affecting eyewitness accuracy is receiver operating characteristic (ROC) analysis^1^ (e.g., Gronlund, Wixted, & Mickes, 2014; Mickes, Flowe, & Wixted, 2012; Wixted & Mickes, 2012). This approach is based on signal detection theory (SDT; see Macmillan & Creelman, 2005), which separates performance into two parameters: response bias versus discriminability. The tradeoff explained by Clark (2012) is best described by SDT as a shift in response bias, whereas the true goal of system variable manipulations is to increase discriminability. Whenever correct and false ID rates are moving in the same direction, even if one is changing to a greater extent, this pattern could be driven by changes in response bias, discriminability, or both. ROC analysis is needed to make this determination, and we will apply this technique to manipulations of lineup composition in order to shed light on the issue of fillers matching the suspect too well.

Four recent studies also applied ROC analysis to manipulations of lineup fairness. Wetmore et al. (2015, 2016) were primarily concerned with comparing showups (presenting a suspect alone rather than with fillers) with simultaneous lineups, but tangentially compared biased with fair simultaneous lineups. A lineup is typically considered biased if the suspect stands out in some way from the fillers. They found that fair lineups yielded higher empirical discriminability compared with biased lineups. Colloff, Wade, and Strange (2016) and Colloff, Wade, Wixted, and Maylor (2017) also found a significant advantage for fair over biased lineups, but defined bias as the presence of a distinctive feature on only one lineup member, and fair as either the presence of the feature on all lineup members or concealed for all members. It is unclear how these distinctive lineups would generalize to more common lineups containing no such obvious distinctive feature. Lastly, Key et al. (2017) found that fair lineups yielded higher empirical discriminability than biased lineups with more realistic stimuli (no distinctive features). However, their target-present and target-absent lineups were extremely biased, containing fillers that matched only one broad characteristic with the suspect (e.g., weight). The official level of fairness was around 1.0 for these biased lineups based on Tredoux’s *E’* (Tredoux, 1998), which ranges from 1 to 6, with 1 representing extreme bias, and 6 representing a very fair lineup. They compared these biased lineups with a target-present and target-absent lineup of intermediate fairness (Tredoux’s *E’* of 3.77 and 3.15, respectively). Our first experiment will add to this literature by evaluating high levels of similarity between fillers and target faces as a test of propitious heterogeneity and the diagnostic feature detection hypothesis (described below). Our second experiment will contribute at a more practical level as the first comparison of suspect-matched and description-matched lineups with ROC analysis.

### Theoretical motivations: propitious heterogeneity and diagnostic feature-detection

Wells, Rydell, and Seelau (1993) argued that lineups should follow the rule of propitious heterogeneity, such that fillers should not be too similar to each other or the suspect (Luus & Wells, 1991; Wells, 1993). At the extreme would be a lineup of identical siblings, such that even a perfect memory of the perpetrator would not help to make a correct ID. Fitzgerald, Oriet, and Price (2015) utilized face morphing software to create lineups with very similar-looking faces. They found that lineups containing highly homogenous faces reduced correct as well as false IDs, thereby creating a tradeoff. More recently, Bergold and Heaton (2018) also found that highly similar lineup members could be problematic, reducing correct IDs and increasing filler IDs. However, neither of these studies applied ROC analysis to address the impact of high similarity among lineup members on empirical discriminability. We will address this issue in the present experiments.

Propitious heterogeneity is a concept with testable predictions (e.g., discriminability will decline at very high levels of filler similarity), but it is not a quantitatively specified theory. In contrast, the diagnostic feature-detection (DFD) hypothesis (Wixted & Mickes, 2014) is a well-specified model that can help explain why it is preferable to have some heterogeneity among lineup members. DFD was initially proffered to explain how certain procedures (e.g., simultaneous lineup versus showup) could increase discriminability. According to this theory, presenting all lineup members simultaneously allows an eyewitness to assess facial features they all share, helping them to determine the more diagnostic features on which to focus when comparing the lineup members to their memory of the perpetrator. However, this should only be useful when viewing a fair lineup in which all members share the general characteristics included in an eyewitness’s description of a perpetrator (e.g., Caucasian man in his 20s with dark hair and a beard). Presenting all members simultaneously (as opposed to sequentially or a showup) allows the eyewitness to quickly disregard these shared features in order to focus on features distinctive to their memory for the perpetrator (see also Gibson, 1969).

DFD theory also predicts that discriminability will be higher for fair over biased simultaneous lineups (Colloff et al., 2016; Wixted & Mickes, 2014). All members of a fair lineup should equivalently match the description of the perpetrator, which should allow the eyewitness to disregard these aspects and focus instead on features that could distinguish between the innocent and the guilty. For example, imagine a perpetrator described as a tall heavy-set Caucasian man with dark hair, a beard, and large piercing eyes. Police would likely ensure that all fillers in the lineup match the general characteristics such as height, weight, race, hair color, and that all have a beard. However, the distinctive eyes would be more difficult to replicate. Therefore, when an eyewitness views a simultaneous lineup, he or she should discount the diagnosticity of these broad characteristics, thereby focusing on internal facial features such as the eyes to make their ID. This process, according to DFD theory, should increase discriminability. In contrast, if the only lineup member with a beard is the suspect (innocent or guilty), the lineup would be biased, and an eyewitness might base their ID largely on this distinctive but nondiagnostic feature. Doing so would reduce discriminability.

It is important to note that there is an important distinction between theoretical and empirical discriminability (see Wixted & Mickes, 2018). DFD predicts changes in theoretical discriminability (i.e., underlying psychological discriminability), which involves latent memory signals affecting decision-making in the mind of an eyewitness. Empirical discriminability is the degree to which eyewitnesses can place innocent and guilty suspects into their appropriate categories. Our experiments will focus on empirical discriminability, which is more relevant for real-world policy decisions (e.g., Wixted & Mickes, 2012, 2018). Empirical discriminability can be used to test the DFD hypothesis because “theoretical and empirical measures of discriminability usually agree about which condition is diagnostically superior” (Wixted & Mickes, 2018, p. 2). In other words, the goal of our experiments is to utilize a theory of underlying psychological discriminability to make predictions about empirical discriminability. Other researchers have noted that it is critical to ground eyewitness ID research in theory (e.g., Clark, Benjamin, Wixted, Mickes, & Gronlund, 2015; Clark, Moreland, & Gronlund, 2014).

The four ROC studies mentioned above (Colloff et al., 2016, 2017; Key et al., 2017; Wetmore et al., 2015) have provided some support for DFD theory by comparing biased with fair lineups. We instead test another prediction that can be derived from the theory: lineups at the highest levels of similarity between fillers and suspect will actually reduce empirical discriminability. In other words, when fillers are too similar to the suspect, potentially diagnostic features are eliminated, which will reduce discriminability according to DFD theory. Similarly, Luus and Wells (1991) predicted that diagnosticity would decline as fillers become more and more similar to each other and the suspect, and Clark, Rush, and Moreland (2013) predicted diminishing returns as filler similarity increases, based on WITNESS model (Clark, 2003) simulations.

We addressed this issue of high filler similarity first in an experiment with computer-generated faces for experimental control. We then conducted a more ecologically valid mock-crime experiment with real faces to test the issue of high filler similarity in the context of description-matched versus suspect-matched fillers. Matching fillers to the suspect could increase the overall level of similarity among lineup members too much (Wells, 1993; Wells et al., 1993), reducing empirical discriminability. If this is the case, we would minimally expect that the similarity ratings between match-to-suspect fillers and the target should be higher than those between match-to-description fillers and the target (Tunnicliff & Clark, 2000). As described below (Experiment 2), we addressed this and also compared description-matched and suspect-matched lineups in ROC space to determine effects on empirical discriminability. There is still much debate in the literature regarding the benefits of matching fillers to description versus suspect (see, e.g., Clark et al., 2013; Fitzgerald et al., 2015). To our knowledge, we are the first to investigate which approach yields higher empirical discriminability. Moreover, despite the historical advocacy for a description-matched approach, to date there are few direct tests of description-matched versus suspect-matched fillers. Lastly, Clark et al. (2014) found that the original accuracy advantage for description-matched fillers has declined over time. One of our goals is to determine if the advantage is real.

## Experiment 1

We utilized FACES 4.0 (IQ Biomatrix, 2003) to tightly control all stimuli in our first experiment.^2^ This program allows for the creation of simple faces based on various combinations of internal (e.g., eyes, nose, mouth) and external (e.g., hair, head shape, chin shape) facial features. The FACES software is commonly used by police agencies (see www.iqbiometrix.com/products_faces_40.html), and has also been used successfully by eyewitness researchers (e.g., Flowe & Cottrell, 2010; Flowe & Ebbesen, 2007), yielding lineup ID results paralleling results from real faces. Moreover, there is some evidence that FACES are processed similarly to real faces, at least to a degree (Wilford & Wells, 2010; but see Carlson, Gronlund, Weatherford, & Carlson, 2012). Regardless of the artificial nature of these stimuli, we argue that the experimental control they allow in terms of both individual FACE creation as well as lineup creation provides an ideal testing ground for theory. Specifically, with FACES we can precisely control the homogeneity of facial features among lineup members, and then work backward from this extreme level to provide direct tests of propitious heterogeneity and the DFD hypothesis.

**Fig. 1:**
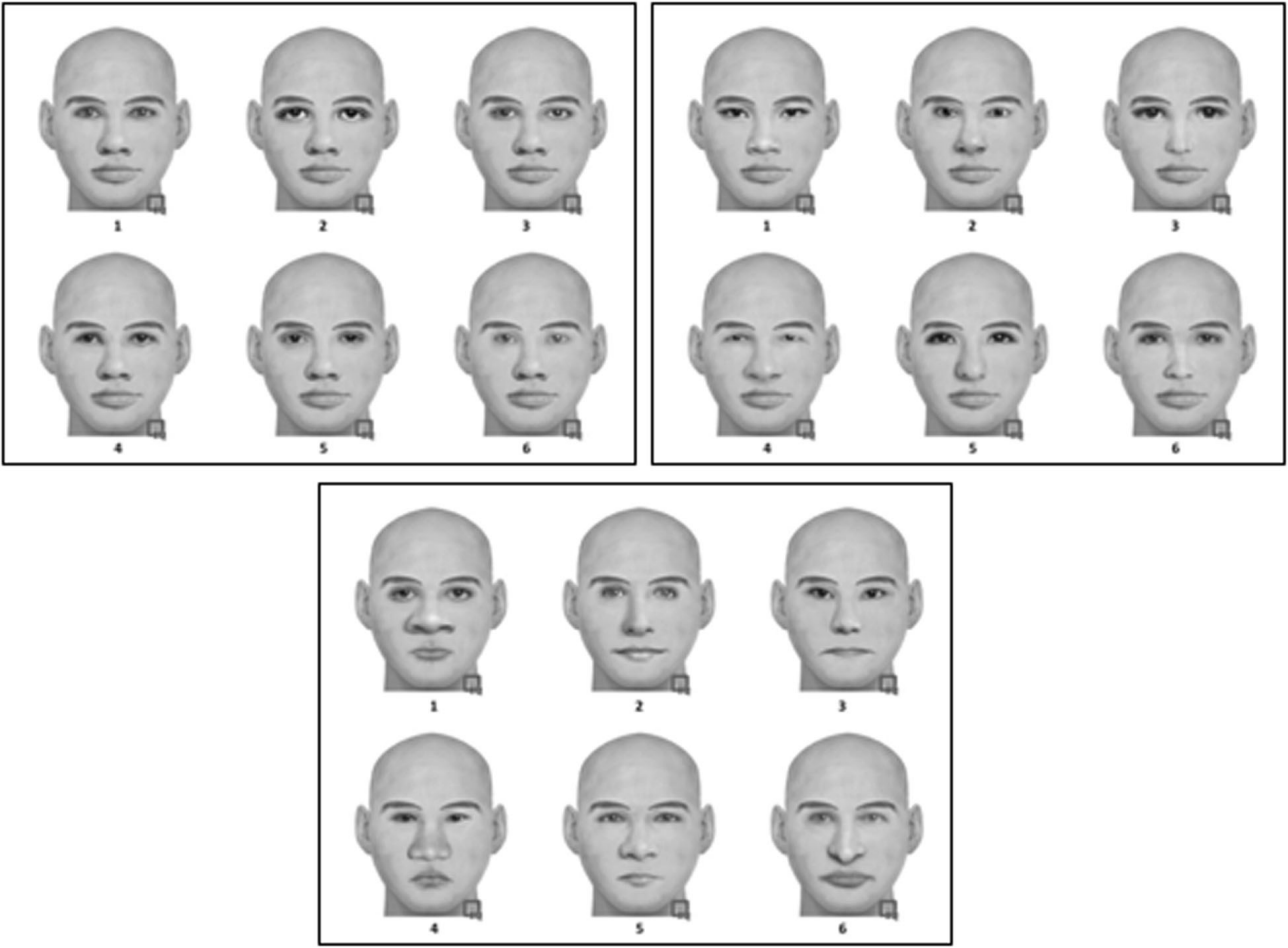
Example lineups from Experiment 1 composed of facial stimuli from FACES 4.0. Only the eyes vary in the top left, the eyes and nose vary in the top right, and eyes, nose, and mouth vary in the bottom

Our participants viewed three types of FACES. In one condition, all FACES in all lineups were essentially target clones, except for one feature that was allowed to vary (the eyes, nose, or mouth; see Fig. 1 for examples). Therefore, participants could base their decision on just one feature rather than the entire FACE. The other two conditions varied two versus three features, respectively. DFD theory predicts that discriminability should increase as participants can base their ID decision on more features that discriminate between guilty and innocent suspects. Therefore, we predicted that empirical discriminability would be best when three features vary, followed by two features, and worst when only one feature varies across FACES in each lineup.

The theoretical rationale is presented in Table 1, which is adapted from Table 1 of Wixted and Mickes (2014). Whereas they were interested in comparing showups with simultaneous lineups, here we present three levels of simultaneous lineups that differ only in the number of features that vary across all fillers. As will be described below, we did not have a designated innocent suspect, but the logic is the same, so we will continue with the “Innocent Suspect” label from Wixted and Mickes. Focus first on the Guilty Suspect rows. Following Wixted and Mickes, and based on signal detection theory, we assume that the target (guilty suspect) was encoded with memory strength values of *M* = 1 and *SD* = 1.22 (so, variance approximately = 1.5 in the table). This, of course, is the case regardless of the fillers, so this remains constant for every lineup type and feature manipulated in a lineup (f1, f2, f3). These three features (f1–3) are the only source of variance (i.e., potentially diagnostic information) in the lineup. If only one feature varies, this means that all fillers (for both target-present and target-absent lineups) are identical to the target except for one feature (eyes, nose, or mouth in our experiments). If two features vary, then all fillers are identical to the target except for two features; if three features vary, then all fillers are identical to the target except for three features.

**Table 1:** Memory strength values of three facial features that are summed to yield an aggregate memory strength value for a face in a simultaneous lineup (adapted from Wixted & Mickes, 2014)

Filler similarity	Suspect	Parameter	f1	f2	f3	Σ	d_a_
One feature varies	Innocent	μ_innocent_	1	1	0	2	0.48
		σ^2^_innocent_	1.5	1.5	1	4	
	Guilty	μ_guilty_	1	1	1	3	
		σ^2^_guilty_	1.5	1.5	1.5	4.5	
Two features vary	Innocent	μ_innocent_	1	0	0	1	1
		σ^2^_innocent_	1.5	1	1	3.5	
	Guilty	μ_guilty_	1	1	1	3	
		σ^2^_guilty_	1.5	1.5	1.5	4.5	
Three features vary	Innocent	μ_innocent_	0	0	0	0	1.55
		σ^2^_innocent_	1	1	1	3	
	Guilty	μ_guilty_	1	1	1	3	
		σ^2^_guilty_	1.5	1.5	1.5	4.5	

f1, f2, and f3 represent the eyes, nose, and mouth, respectively

Critically, the Innocent Suspect rows change across these levels of similarity, reflecting featural overlap with the guilty suspect. When only one feature varies in the lineup, only f3 differs between fillers and guilty suspect, and f1 and f2 are identical. For example, this occurs when the participant in this condition sees that the lineup is entirely composed of clones except that all lineup members have a different mouth. This is the case for target-present (TP) and target-absent (TA) lineups, making the mouth diagnostic of suspect guilt (only one lineup member serves as the target with the correct mouth). This is represented by the top rows of Table 1: One Feature Varies. For that feature (f3; e.g., mouth), the memory strength values for the innocent suspect are *M* = 0 and *SD* = 1 (see Wixted & Mickes, 2014). Moving down to the next lineup type, two features vary, so now the memory strength values for the innocent suspect are set to *M* = 0 and *SD* = 1 for f2 as well as f3. This would be the case if, for example, both the nose and the mouth differ between innocent suspect (i.e., all fillers, as in our experiments) and guilty suspect. Finally, the bottom rows represent lineups in which all three features vary (eyes, nose, and mouth), which decreases the overlap between innocent and guilty suspects even further (i.e., between fillers and the target). As can be seen in the far-right column, underlying psychological discriminability is expected to increase as more features are diagnostic of suspect guilt in the lineup, based on the unequal variance signal detection model:

$$ {d}_a=\frac{\upmu_{guilty}-{\mu}_{innocent}}{\sqrt{\left({\upsigma}_{guilty}^2+{\upsigma}_{innocent}^2\right)/2}} $$

We assessed whether empirical discriminability would increase as more facial features in each of the fillers differ from the target (i.e., as more features are present that are diagnostic of suspect guilt). In other words, as the fillers look less and less like the target (with more features allowed to vary), participants should be better able to identify the target and reject fillers.

### Method

#### Participants

Students from the Texas A&M University – Commerce psychology department subject pool served as participants (*N* = 100). Based on the within-subjects design described below, this sample size allowed us to obtain 300 data points per cell. Although some more recent eyewitness studies applying ROC analysis to lineup data have included around 500 or more participants or data points per cell (e.g., Seale-Carlisle, Wetmore, Flowe, & Mickes, 2019) other studies have shown that 100–200 is sufficient (e.g., 100–130/cell in Carlson & Carlson, 2014; around 150/cell in Mickes et al., 2012), and so both experiments in this paper included at least 200 data points per experimental cell. We obtained approval from the university’s institutional review board for both experiments in this paper, and informed consent was provided by each participant at the beginning of the experiment.

#### Materials

We utilized the FACES 4.0 software (IQ Biometrix, 2003) to create our stimuli (see Fig. 1 for examples). No face had any hair or other distinguishing external characteristics; all shared the same external features as seen in Fig. 1. The only features that varied were the eyes, nose, and/or mouth. The critical independent variable, manipulated within subjects, was how many of these features varied in a given lineup. Under one condition, only one of these features varied in a given lineup. For example, all members of a given lineup were clones except that each would have different eyes. Therefore, participants could base their lineup decision (for both TP and TA lineups) on the eyes alone. The same logic applied to lineups with only the mouth being different among the lineup members, as well as those in which only the nose varied. However, when encoding each face prior to the lineup, participants did not know which of the three features (or how many features, as this was manipulated within subjects) would vary in the upcoming lineup. Under another condition, two of these three features varied in a given lineup, thereby providing participants with more featural information on which to base their ID decision (again, for both TP and TA lineups). Lastly, all three features varied under the third condition of this independent variable. Each target was randomly assigned to a position during creation of the TP lineups (see Carlson et al., 2019, for the importance of randomizing or counter-balancing suspect position), and there was no designated innocent suspect in TA lineups.

#### Procedure and design

Participants took part in a face recognition paradigm with 18 blocks, and research has shown that lineup responses across multiple trials are similar to single-trial eyewitness ID paradigms (Mansour, Beaudry, & Lindsay, 2017). Both target presence (TP vs. TA lineup) and the number of diagnostic features in each lineup (1–3) were manipulated within subjects. Each of the 18 blocks contained the same general procedure: encoding of a single FACE, distractor task, then lineup. For each encoding phase, we simply presented the target FACE for 1 s in the middle of the screen. The distractor task in each block was a word search puzzle on which participants worked for 1 min between the encoding and lineup phase of each block. The final part of each block was the critical element: a simultaneous lineup of six FACES presented in a 2 × 3 array, and participants were instructed to identify the target presented earlier in that block, which may or may not be present. They could choose one of the six lineup members or reject the lineup. After their decision, they entered their confidence on an 11-point scale (0–100% in 10% increments), and then the next block automatically began. There were three blocks dedicated to each of the six experimental cells: 1) TP vs TA lineup with one feature varying; 2) TP vs TA lineup with two features varying; and 3) TP vs TA lineup with three features varying. Each participant viewed a randomized order of these blocks.

### Results

See Table 2 for all correct, false, and filler IDs, along with lineup rejections. We will first describe the results of ROC analysis, followed by TP versus TA lineup data separately (Gronlund & Neuschatz, 2014). We applied Bonferroni correction (α = .05/3 = .017) to control Type I error rate due to multiple comparisons.

**Table 2:** Number of identifications and rejections from Experiment 1

Condition	Target-present lineups			Target-absent lineups	
	Correct ID rate	Filler ID rate	Rejection rate	Filler ID rate	Rejection rate
One feature varies	0.65	0.25	0.1	0.72	0.28
Two features vary	0.76	0.16	0.08	0.35	0.65
Three features vary	0.73	0.08	0.19	0.19	0.81

*ID* identification

#### ROC analysis

**Fig. 2:**
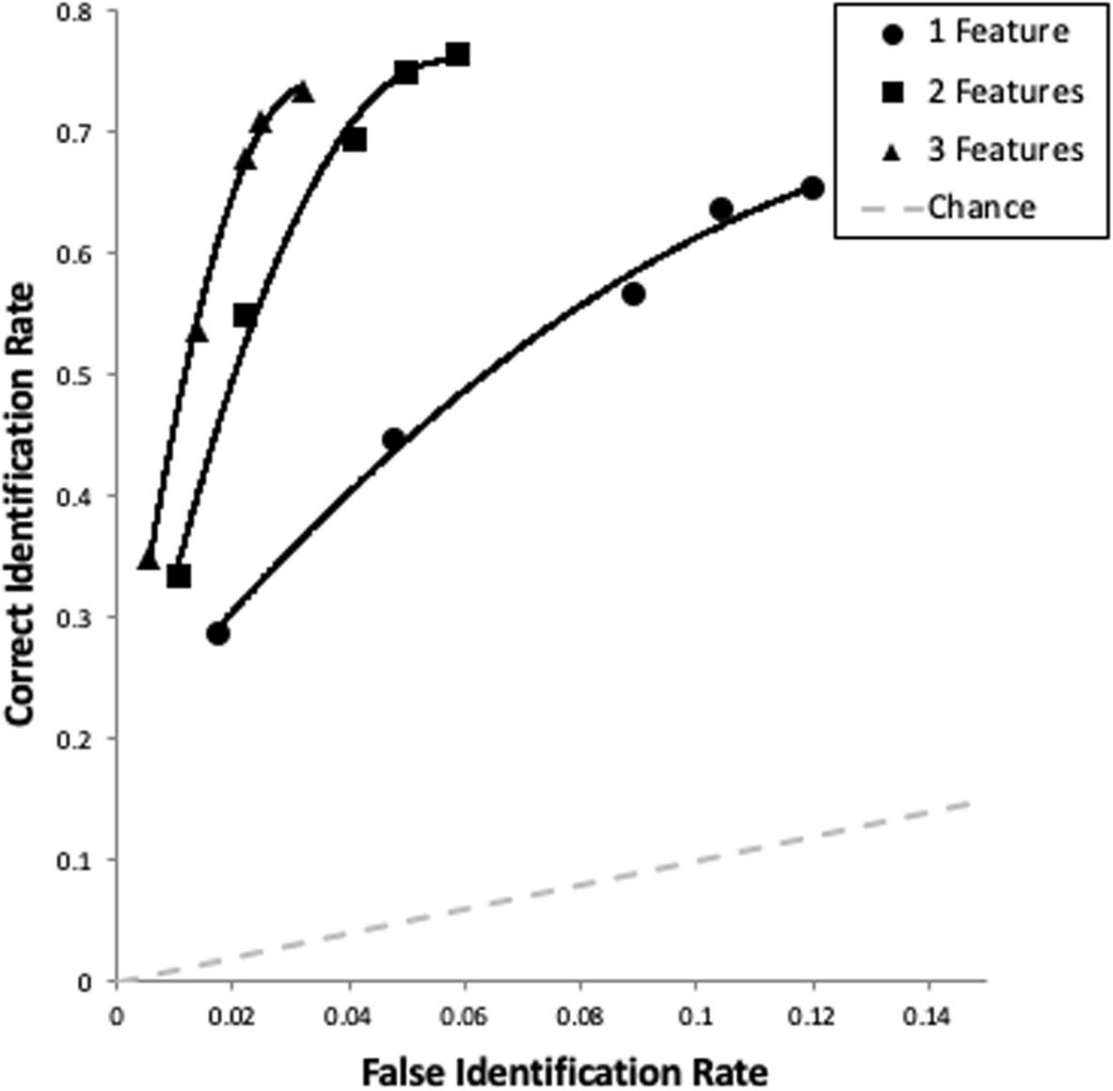
ROC data from Experiment 1. The curves drawn through the empirical data points are not based on model fits, but rather are simple trendlines drawn in Excel. The correct ID rate on the *y* axis is the proportion of targets chosen from the total number of target-present lineups in a given condition. The false ID rate on the *x* axis is the proportion of all filler identifications from the total number of target-absent lineups in a given condition (as we had no designated innocent suspects), divided by the nominal lineup size (six) to provide an estimated innocent suspect ID rate

It is important to determine how our manipulations affected empirical discriminability independently of a bias toward selecting any suspect (whether guilty or innocent), which is what ROC analysis is designed to accomplish (e.g., Gronlund et al., 2014; Rotello & Chen, 2016; Wixted & Mickes, 2012). As shown in Fig. 2, each condition results in a curve in ROC space constructed from correct and false ID rates across levels of confidence. In order to be comparable to the correct ID rates of targets from TP lineups, the total number of false IDs from TA lineups were divided by the number of lineup members (6) to calculate false ID rates, which is a common approach in the literature when there is no designated innocent suspect (e.g., Mickes, 2015). All data from a given condition reside at the far-right end of its curve, and then the curve extends to the left first by dropping participants with low levels of confidence. Thus, the second point from the far right of each curve excludes IDs that were supported by confidence of 0–20%, then the third point excludes these IDs as well as those supported by 30–40% confidence. This process continues for each curve until the far-left point represents only those IDs supported by the highest levels of confidence (here 90–100%). Confidence thereby serves as a proxy for the bias for choosing any suspect (regardless of guilt), with the most conservative suspect IDs residing on the far left, and the most liberal on the far right.

The level of empirical discriminability for each curve is determined with the partial area under the curve (pAUC; Robin et al., 2011). The farther a curve resides in the upper-left quadrant of the space, the greater the empirical discriminability. The pAUC rather than full AUC is calculated because TA filler IDs are divided by six, thereby preventing false ID rate on the *x* axis from reaching 1.0. Finally, each pair of curves can be compared with *D* = (pAUC1 – pAUC2)/*s*, where *s* is the standard error of the difference between the two pAUCs after bootstrapping 10,000 times (see Gronlund et al., 2014, for a tutorial).

As seen in Fig. 2, there was no significant difference between three features (pAUC = .088 [.079–.097]) and two features (pAUC = .086 [.075–.096]), *D* = 0.46, not significant (ns). However, having multiple diagnostic features boosted empirical discriminability beyond just one feature (pAUC = .061 [.050–.072]): (a) two features were better than one, *D* = 3.98, *p* < .001, and (b) three features were better than one, *D* = 4.58, *p* < .001. This pattern largely supports both the concept of propitious heterogeneity and the DFD hypothesis.

#### Separate analyses of TP and TA lineups

The number of diagnostic features in each lineup significantly impacted correct IDs, Wald (2) = 9.48, *p* = .009. Chi-square analyses revealed that, though there was no difference between two and three diagnostic features (χ^2^ = 1, *N* = 600) = 0.72, ns), we did confirm that having just one diagnostic feature yielded fewer correct IDs compared with both two features (χ^2^ (1, *N* = 600) = 8.79, *p* = .002, ϕ = .12) and marginally fewer compared with three features (χ^2^ (1, *N* = 600) = 4.52, *p* = .02, ϕ = .09).

False IDs (of any lineup member from TA lineups) were affected even more so by the number of diagnostic features, Wald (2) = 159.59, *p* < .001. Participants were much more likely to choose lineup members from TA lineups when only one feature varied compared with two features (χ^2^ (1, *N* = 600) = 81.10, *p* < .001, ϕ = .37) or three features (χ^2^ (1, *N* = 600) = 167.69, *p* < .001, ϕ = .53). There were also more false alarms when two features varied compared with three, χ^2^ (1, *N* = 600) = 19.33, *p* < .001, ϕ = .18. In summary, unsurprisingly, the more the lineup members matched the target (i.e., with fewer features varying across members), the more participants chose these faces.

### Discussion

In support of other research investigating lineups of high filler similarity (e.g., Fitzgerald et al., 2015), these results indicate that lineups containing very similar fillers could be problematic, as they tended to lower ID accuracy (see also simulations by Clark et al., 2013). We went a step beyond the literature to show with ROC analysis that empirical discriminability declines at the upper levels of filler similarity. Allowing more features to vary among lineup members generally increased accuracy. These preliminary findings support the principle of propitious heterogeneity (e.g., Wells et al., 1993) and the DFD hypothesis (Wixted & Mickes, 2014).

## Experiment 2

Here, our goal was to extend the logic of the first experiment to an issue of more ecological importance than lineups of extremely high levels of featural homogeneity, which would not occur in the real world. Instead, we focused on whether police should select fillers based on matching a suspect’s description or a suspect himself. Both should lead to fair lineups that yield higher empirical discriminability compared with showups (Wetmore et al., 2015; Wixted & Mickes, 2014) or compared with biased lineups (e.g., Key et al., 2017). However, suspect-matched lineups could have fillers that are more similar to the suspect than description-matched lineups because each filler is selected based directly on the suspect’s face. Features that otherwise would be diagnostic of guilt could thereby be replicated in TP lineups, which could reduce correct ID rate. A greater overlap of diagnostic features would also reduce discriminability according to the DFD hypothesis. In this experiment, we compared suspect-matched with description-matched lineups to determine which should be recommended to police. Others have compared these filler selection methods (e.g., Lindsay, Martin, & Webber, 1994; Luus & Wells, 1991; Tunnicliff & Clark, 2000), but we make two contributions beyond this prior research: 1) we will assess which method yields higher empirical discriminability; and 2) we will test a theoretical prediction based on propitious heterogeneity and the DFD hypothesis that higher similarity between fillers and suspect in suspect-matched lineups will contribute to lower empirical discriminability compared with description-matched lineups.

### Method

#### Participants

As mentioned above, based on eyewitness ID studies utilizing ROC analysis (e.g., Carlson & Carlson, 2014; Mickes et al., 2012), we sought a minimum of 200 participants for each lineup that we created. As described below, we created nine lineups, requiring a minimum of 1800 participants. We utilized SurveyMonkey to offer this experiment to a nationwide sample of participants (*N* = 2159) in the United States. We dropped 194 participants for providing incomplete data or failing to answer our attention check question correctly, leaving 1965 for analysis (see Table 3 for demographics).

**Table 3:** Demographics for Experiment 2

	*n*
Sex	
Male	857
Female	1108
Age (years)	
18–29	609
30–44	513
45–60	556
Over 60	276
No response	11
Ethnicity	
African–American	151
Caucasian/White	1601
Hispanic/Latino	102
Asian	33
Other	63
No response	15
*N*	1965

#### Materials

##### Mock crime video

We used a mock crime video from Carlson et al. (2016), which presents a woman sitting on a bench surrounded by trees in a public park. A male perpetrator^3^ emerges from behind a large tree in the right of the frame, approaches the woman slowly, and grabs her purse before running away. He is visible for 10 s, and is approximately 3 m from the camera when he emerges from behind the tree, and about 1.5 m away when he reaches the victim. A photo of the perpetrator taken a few days later was used as his lineup mugshot.

##### Description-matched lineups

In order to create description-matched lineups, we first needed a modal description for the perpetrator. A group of undergraduates (*N* = 54^4^) viewed the mock crime video and then answered six questions regarding the perpetrator’s physical characteristics. We used the most frequently reported descriptors to create the modal description (white male, 20–30 years old, tall, short hair, stubble-like facial hair). We gave this description to four research assistants (none of whom ever saw the mock crime video or perpetrator mugshot) and asked each of them to pick 20 matches from various public offender mugshot databases (e.g., State of Kentucky Department of Corrections) to create a pool of 80 description-matched fillers.

We randomly selected 10 mugshots from the description-matched pool to serve as fillers in the two description-matched TP lineups. In order to avoid stimulus-specific effects lacking generalizability (Wells & Windschitl, 1999), we used two designated innocent suspects who were randomly selected from the description-matched pool. To further increase generalizability, we then created two TA lineups for each of these two innocent suspects, for a total of four description-matched TA lineups. Twenty additional mugshots were randomly selected from the pool to serve as fillers in these lineups.

To assess lineup fairness, we presented an independent group of undergraduates (*N* = 28) with each lineup and they chose the member that best matched the perpetrator’s modal description. We used these data to calculate Tredoux’s *E’* (Tredoux, 1998), which is a statistic ranging from 1 (very biased) to 6 (very fair): TP Lineup 1 (3.09), TP Lineup 2 (4.17), Lineup 1 for Innocent Suspect 1 (4.08), Lineup 2 for Innocent Suspect 1 (5.09), Lineup 1 for Innocent Suspect 2 (4.04), and Lineup 2 for Innocent Suspect 2 (4.36).

##### Suspect-matched lineups

We started by providing the perpetrator’s mugshot to a new group of four research assistants, asking each of them to pick 20 matches from the mugshot databases (e.g., State of Kentucky Department of Corrections) to create a pool of 80 suspect-matched fillers. We randomly selected five mugshots from this pool to serve as fillers in the suspect-matched TP lineup. We then randomly selected 49 mugshots from the description-matched pool, which an independent group of undergraduates (*N* = 30) rated for similarity to each of the innocent suspects using a 1 (least similar) to 7 (most similar) Likert scale. The five most similar faces to each innocent suspect served as fillers in their respective suspect-matched TA lineup. We therefore had a total of three suspect-matched lineups: one for the perpetrator and one for each innocent suspect (these are the same two innocent suspects as in the description-matched lineups, as police would never apprehend a suspect because he matches a perpetrator). The same group of 28 participants who reviewed the description-matched lineups also evaluated these lineups for fairness, resulting in Tredoux’s *E’* (Tredoux, 1998) of 3.27 for the TP lineup, 4.45 for TA Lineup 1, and 5.16 for TA Lineup 2. These results are comparable to the description-matched lineups.

According to the prediction of Luus and Wells’ (1991) that a suspect-matched procedure could produce fillers that are too similar to the suspect, similarity ratings should be higher for suspect-matched lineups than for description-matched lineups (see also Tunnicliff & Clark, 2000). This is also necessary according to the DFD hypothesis to create a situation that would lower empirical discriminability. To establish the level of similarity, an independent group of participants (*N* = 50^5^) rated the similarity of the suspect to each of the five fillers in their respective lineups on a 1 (least similar) to 7 (most similar) Likert scale. Indeed, overall mean similarity between each filler and the suspect was higher for suspect-matched lineups (*M* = 2.84, *SD* = 1.26) compared with description-matched lineups (*M* = 2.11, *SD* = 1.20), *t* (49) = 9.05, *p* < .001. This pattern is consistent across both TP (suspect-matched *M* = 3.56, *SD* = 1.39; description-matched *M* = 2.20, *SD* = 1.18; *t*(49) = 9.31, *p* < .001) and TA lineups (suspect-matched *M* = 2.48, *SD* = 1.32; description-matched *M* = 2.07, SD = 1.22; *t*(49) = 5.91, *p* < .001). These patterns, as well as the overall low similarity ratings (all less than mid-point of 7-point Likert scale) are consistent with results from earlier studies (e.g., Tunnicliff & Clark, 2000; Wells et al., 1993).

#### Design and procedure

This experiment conformed to a 2 (filler selection method: suspect-matched vs. description-matched lineup) × 2 (TP or TA lineup) between-subjects factorial design. After informed consent, participants watched the mock crime video followed by another video (about protecting the environment) serving as a distractor for 3 min. After answering a question about the distractor video to confirm that they watched it, each participant was randomly assigned to view a six-person TP or TA simultaneous lineup, containing either suspect-matched or description-matched fillers. All lineups were formatted in a 2 × 3 array, and the position of the suspect was randomized. Each lineup was accompanied with instructions that stated that the perpetrator may or may not be present. Immediately following their lineup decision, participants rated their confidence on a 0%–100% scale (in 10% increments). Finally, they answered an attention check question (“What crime did the man in the video commit?”) as well as demographic questions pertaining to age, sex, and race.

### Results

As with our earlier experiment, we will first present the results of ROC analysis to determine differences in empirical discriminability, followed by logistic regression and chi-square analyses to the TP data separately from the TA data. All reported *p* values are two-tailed. See Table 4 for all ID decisions across all lineups.

**Table 4:** Number of identifications and rejections from Experiment 2

Filler selection method	Lineup	Suspect ID rate	Filler ID rate	Rejection rate
Suspect-matched	TP	.38 (81/214)	.45 (97/214)	.17 (36/214)
	TA1	.17 (33/200)	.37 (73/200)	.47 (94/200)
	TA2	.16 (34/210)	.40 (83/210)	.44 (93/210)
Description-matched	TP1	.57 (120/212)	.23 (48/212)	.21 (44/212)
	TP2	.58 (123/211)	.16 (34/211)	.26 (54/211)
	TA1.1	.22 (60/268)	.41 (111/268)	.36 (97/268)
	TA1.2	.33 (71/212)	.21 (44/212)	.46 (97/212)
	TA2.1	.13 (27/210)	.40 (84/210)	.47 (99/210)
	TA2.2	.16 (36/228)	.40 (91/228)	.44 (101/228)

All TP lineups contained the same target; TP1 and TP2 contained different fillers with the same target

There were two innocent suspects (IS) to increase generalizability. Suspect-Matched TA1 contained IS1 and TA2 contained IS2. Description-Matched TA1.1 and TA1.2 each had different fillers with IS1; TA2.1 and TA2.2 each had different fillers with IS2

*ID* identification, *TP* target-present, *TA* target-absent

#### ROC analysis

**Fig. 3:**
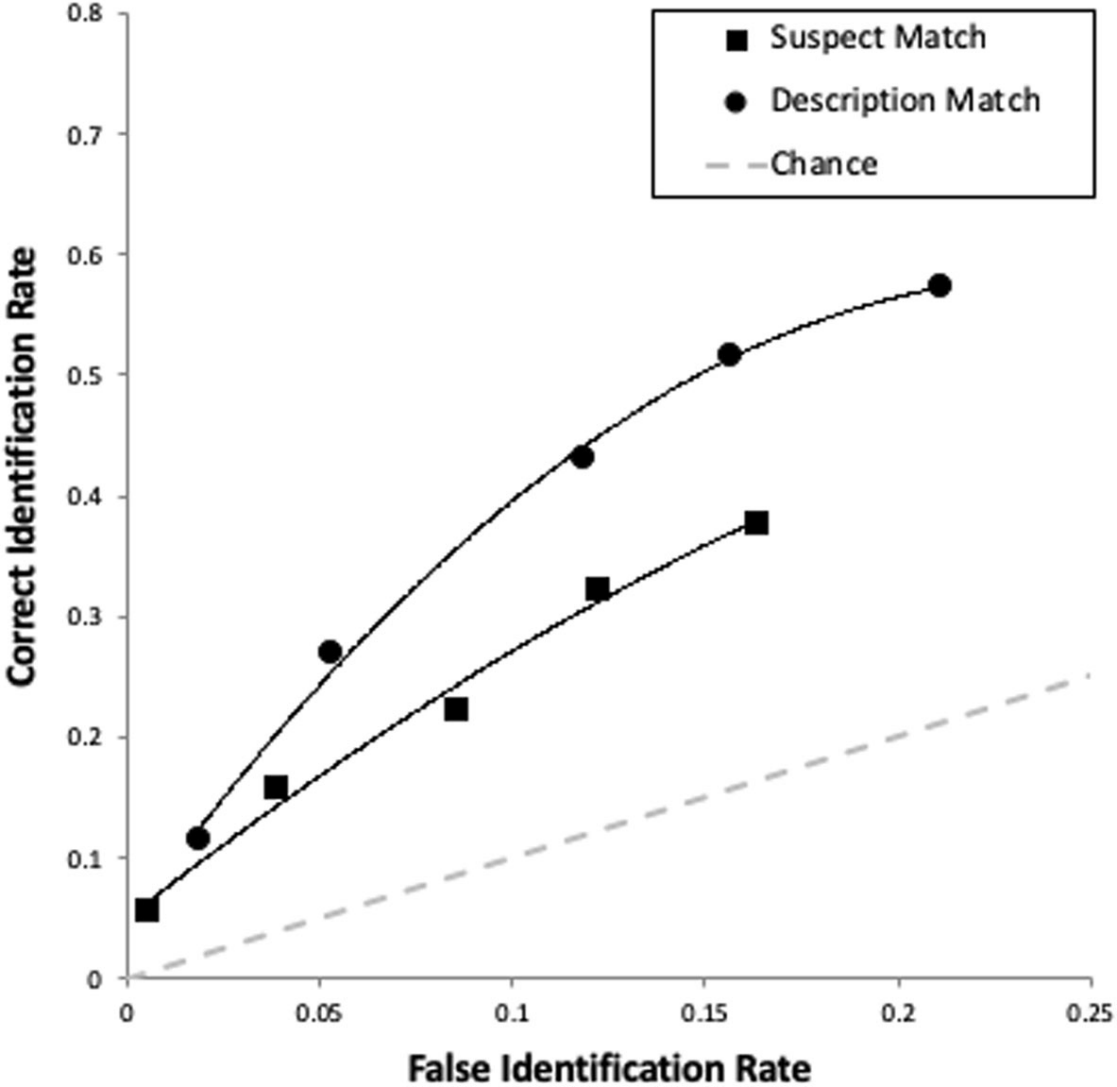
ROC data (with trendlines) from Experiment 2 collapsed over the different description-matched and suspect-matched lineups. The false ID rate on the *x* axis is the proportion of innocent suspect identifications from the total number of target-absent lineups in a given condition

Our primary goal was to determine whether description-matched lineups would increase empirical discriminability compared with suspect-matched lineups. To address this, we compared the description-matched ROC curve with the suspect-matched curve, collapsing over individual lineups (specificity = .84^6^; see Fig. 3). As predicted, matching fillers to description (pAUC = .052 [.045–.059]) increased empirical discriminability compared with matching fillers to suspect (pAUC = .037, [.029–.045]), *D* = 2.61, *p* = .009. As for the bias toward choosing any suspect, description-matched lineups overall induced more liberal suspect choosing (as shown by the longer ROC curve in Fig. 3) compared with the suspect-matched lineups. This effect on response bias replicates other research comparing these two methods of filler selection without ROC analysis (Lindsay et al., 1994; Tunnicliff & Clark, 2000; Wells et al., 1993).

**Fig. 4:**
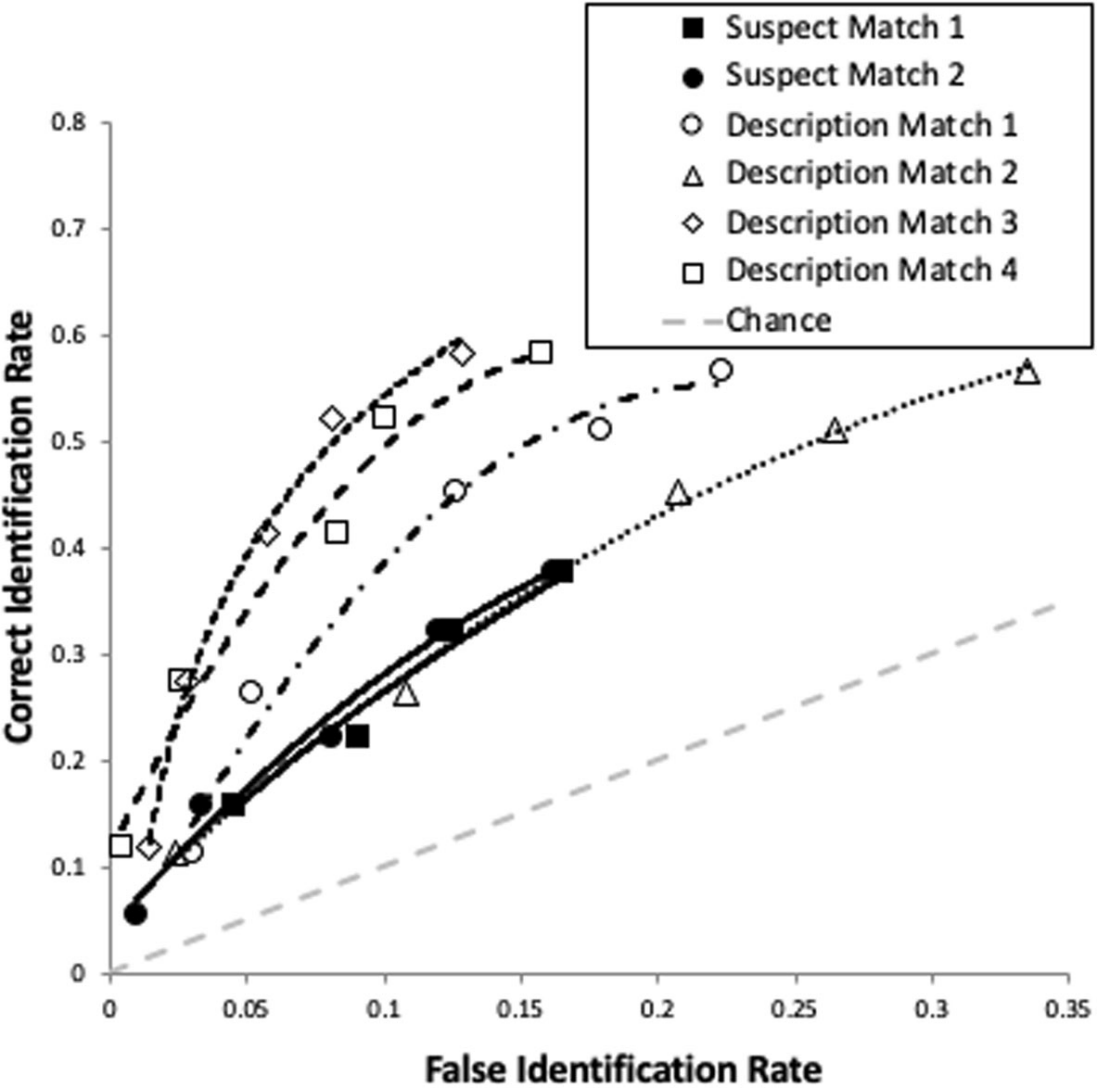
ROC data (with trendlines) for all description-matched and suspect-matched lineups from Experiment 2

In order to address the robustness of the overall effect on empirical discriminability, we then broke down the curves into four description-matched curves and two suspect-matched curves (Fig. 4; specificity = .66). The description-matched curves were based on correct ID rates from the two TP lineups (each with the same target but different description-matched fillers) combined with false alarm rates from four TA lineups (two with fillers matching the description of innocent suspect 1, and two with fillers matching the description of innocent suspect 2). The two suspect-matched curves are based on the correct ID rate from the one suspect-matched TP lineup and the false alarm rates from the two suspect-matched TA lineups (one for innocent suspect 1 and one for innocent suspect 2). See Table 5 for the pAUC of each curve and Table 6 for the comparison between each description-matched and suspect-matched curve (Bonferroni-corrected α = .05/8 = .006). No suspect-matched curve ever increased discriminability compared with a description-matched curve. Rather, two description-matched curves yielded greater discriminability than both suspect-matched curves.^7^

**Table 5:** Results of receiver operating characteristic analysis for Experiment 2

	pAUC	Confidence interval
Suspect match 1	.025	.017–.036
Suspect match 2	.027	.018–.037
Description match 1	.035	.024–.047
Description match 2	.025	.017–.035
Description match 3	.051	.038–.066
Description match 4	.048	.036–.060

*pAUC* partial area under the curve

**Table 6:** Comparison of each suspect-matched lineup with each description-matched lineup from Experiment 2

	Suspect match 1		Suspect match 2	
	*D*	*p*	*D*	*p*
Description match 1	1.40	.16	1.17	.24
Description match 2	0.04	.97	0.27	.79
Description match 3	3.13	.002	2.92	.004
Description match 4	2.90	.004	2.61	.01

#### Separate analyses of TP and TA lineups

We begin with the correct IDs. As a reminder, there was one suspect-matched TP lineup and two description-matched TP lineups, so Bonferroni-corrected α = .05/2 = .025. The full logistic regression model was significant, showing that there were more correct IDs for the description-matched lineups compared with the suspect-matched lineup, Wald (2) = 21.57, *p* < .001. This pattern was supported by follow-up chi-square tests comparing the suspect-matched lineup with: (a) Description-Matched TP1, χ^2^ (1, *N* = 426) = 15.03, *p* < .001, ϕ = .19; and (b) Description-Matched TP2, χ^2^ (1, *N* = 425) = 17.79, *p* < .001, ϕ = .21. As for filler IDs from TP lineups, the full logistic regression model was again significant, Wald (2) = 46.82, *p* < .001. The filler ID rate was higher for the suspect-matched lineup compared with both Description-Matched TP1, χ^2^ (1, *N* = 426) = 24.41, *p* < .001, ϕ = .24, and Description-Matched TP2, χ^2^ (1, *N* = 425) = 42.52, *p* < .001, ϕ = .32. Lastly, the model for TP lineup rejections was not significant, Wald (2) = 4.89, *p* = .087.

Turning to the TA lineups, there were two suspect-matched (each based on its own innocent suspect) and four description-matched (the same two innocent suspects × 2 sets of fillers each). The full model comparing false IDs across all six lineups was significant, Wald (5) = 36.47, *p* < .001. A follow-up chi-square found that the false ID rate was lower for the suspect-matched lineups compared with the description-matched lineups overall, χ^2^ (1, *N* = 1328) = 4.12, *p* = .042, ϕ = .06. There was no difference in filler IDs or correct rejections. The next step was to compare each suspect-matched lineup with each description-matched lineup to determine the consistency of the pattern of false IDs (Bonferroni-corrected α = .05/8 = .006). Of the eight comparisons, only two were significant: (a) Suspect-Matched TA1 yielded fewer false IDs than Description-Matched TA1.2, χ^2^ (1, *N* = 412) = 15.74, *p* < .001, ϕ = .20; and (b) Suspect-Matched TA2 yielded fewer false IDs than Description-Matched TA1.2, χ^2^ (1, *N* = 422) = 16.89, *p* < .001, ϕ = .20. As can be seen in Table 4, Description-Matched TA1.2 had a higher false ID rate than any other TA lineup, which drove the overall effect of more false IDs for description-matched over suspect-matched lineups. The more consistent finding was no difference in false IDs between the two filler selection methods. We reviewed these lineups in light of these results, and could not determine why the false ID rate was higher for TA1.2, as the innocent suspect does not appear to stand out from the fillers. In fact, this lineup had the highest level of fairness (*E’* = 5.09) compared with the other description-matched TA lineups (4.08, 4.04, and 4.36). This indicates that Tredoux’s *E’*, and likely other lineup fairness measures that are based on a perpetrator’s description, could inaccurately diagnose a lineup’s level of fairness. This point has recently been supported by a large study comparing several methods of evaluating lineup fairness (Mansour, Beaudry, Kalmet, Bertrand, & Lindsay, 2017).

#### Confidence-accuracy characteristic analysis

Discriminability is an important consideration when it comes to system variables, such as filler selection method, but the reliability of an eyewitness’s suspect identification, given their confidence, is also critical. Whereas ROC analysis is ideal for revealing differences in discriminability, some kind of confidence-accuracy characteristic (CAC) analysis is needed to investigate reliability (Mickes, 2015). In other words, to a judge and jury evaluating an eyewitness ID from a given case, one piece of information will be the filler selection method used by police when constructing the lineup. Another piece of information will be the eyewitness’s confidence in their lineup decision, which studies have shown has a strong relationship to the accuracy of the suspect ID given that it is immediately recorded after the suspect ID, and the lineup was conducted under good conditions (e.g., double-blind administrator and a fair lineup; see Wixted & Wells, 2017). Recent studies have supported a strong CA relationship across various manipulations, such as weapon presence during the crime (Carlson et al., 2017), amount of time to view the perpetrator during the crime (Palmer, Brewer, Weber, & Nagesh, 2013), and lineup type (simultaneous versus sequential; Mickes, 2015). The present experiment allowed us to test suspect- versus description-matched filler selection methods in terms of the CA relationship. We had no explicit predictions regarding this comparison, but provide the CAC analysis due to its applied importance.

**Fig. 5:**
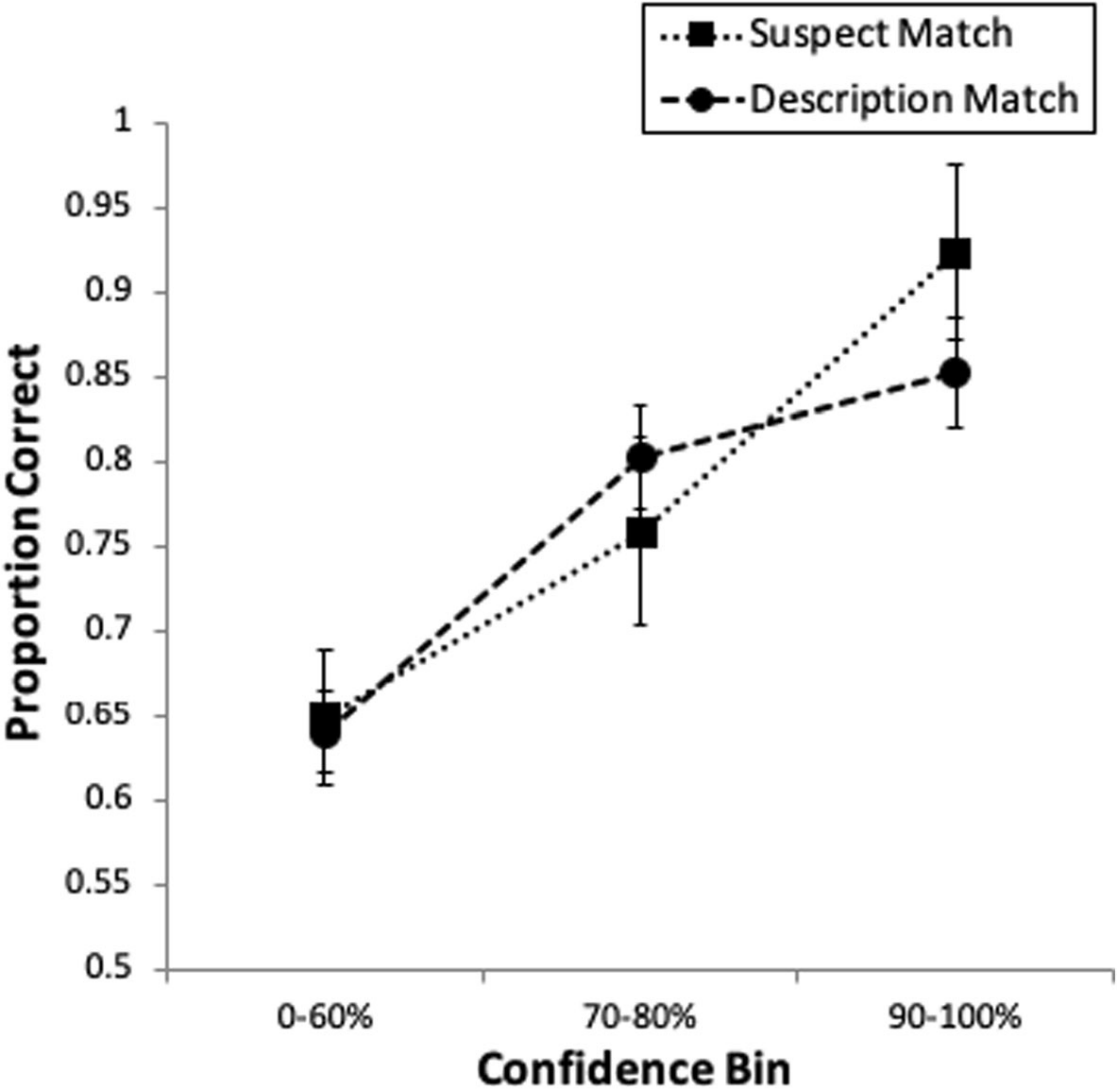
CAC data from Experiment 2. The bars represent standard errors. Proportion correct on the *y* axis is #correct IDs/(#correct IDs + #false IDs)

As can be seen in Fig. 5, there is a strong CA relationship across both filler selection methods. The *x* axis represents three levels of confidence (0–60% for low, 70–80% for medium, and 90–100% for high), which is typically broken down in this way for CAC analysis (see Mickes, 2015). The *y* axis represents the conditional probability (i.e., positive predictive value): given a suspect ID, what is the likelihood that the suspect was guilty, represented as guilty suspect IDs/(guilty suspect IDs + innocent suspect IDs). Two results are of note from Fig. 5: 1) confidence is indicative of accuracy, such that both curves have positive slopes; and 2) suspect IDs supported by high confidence are generally accurate (85% or higher).

### Discussion

This is the first experiment (to our knowledge) to address which method of filler selection, description- versus suspect-match, yields the highest empirical discriminability. We found that matching fillers to description appears to be the preferred approach, as it increased the ability of our participant eyewitnesses to sort innocent and guilty suspects into their proper categories. This was the case when collapsing over all individual lineups and, when making all pairwise comparisons between description- and suspect-matched lineups, we found that no suspect-matched lineup ever increased discriminability beyond a description-matched lineup. Rather, description-matched lineups were either better than, or equivalent to, suspect-matched lineups. We discuss the potential reasons for the overall advantage for description-matched lineups below.

## General discussion

We supported two theories from the eyewitness identification literature: propitious heterogeneity (e.g., Wells et al., 1993) and diagnostic feature-detection (DFD; Wixted & Mickes, 2014) by showing that empirical discriminability decreases as fillers become too similar to each other and the suspect. Our first experiment demonstrated this phenomenon with computer-generated faces that we could manipulate to precisely control levels of similarity among lineup members. Experiment 2 extended this effect to the real-world issue of filler selection, showing that police should match fillers to the description of a perpetrator rather than to a suspect. However, this recommendation is not without its caveats, such as the level of detail of a particular eyewitness’s description.

This issue of specificity of the description for description-matched lineups is a question ripe for empirical investigation. To our knowledge, there has been no research on the influence of description quality (i.e., number of fine-grained descriptors) on the development of lineups and resulting empirical discriminability. Based on our findings, we would predict an inverted U-shaped function on empirical discriminability, such that eyewitnesses would perform best on description-matched lineups with fillers matched to a description that is not too vague (see Lindsay et al., 1994) and also not too specific. The former could yield biased lineups, whereas the latter could yield lineups with fillers that are too similar to the perpetrator, akin to the suspect-matched lineups that we tested. We encourage researchers to investigate this important issue of descriptor quality and eyewitness ID. Minimally, this research would address the issue of boundary conditions for description- versus suspect-matched lineups. At what point are suspect-matched lineups superior? Surely, if the description of the perpetrator is sufficiently vague, discriminability would be higher for suspect-matched lineups, but this is an empirical question.

Other than filler similarity, there is at least one more explanation for the reduction in empirical discriminability that we found for suspect-matched lineups. In the basic recognition memory literature, within-participant variance in responses has been shown to reduce discriminability (e.g., Benjamin, Diaz, & Wee, 2009). Mickes et al. (2017) found that variance among eyewitness participants can reduce empirical discriminability in a similar manner. Their variance was created by different instructions prior to the lineup (to induce conservative versus liberal choosing), which could have been interpreted or adhered to differently across participants. Similarly, suspect-matched lineups have an additional source of variance compared with description-matched lineups, which could have contributed to the lowering of empirical discriminability for suspect-matched lineups. For description-matched lineups, all fillers are selected based on matching a single description. Assuming the description is not too vague, this should limit the overall variance across fillers. In contrast, suspect-matched fillers are matched to the target for TP lineups and to a completely different individual (the innocent suspect) for TA lineups. This would likely add variance to the similarity of fillers across these two conditions, thereby lowering empirical discriminability. However, although alternative explanations such as criterial variability are always possible, it is important to note that the DFD theory predicted our results in advance, making it a particularly strong competitor with other potential explanations of the effect of lineup fairness and filler similarity on empirical discriminability. This also illustrates the importance of theory-driven research for the field of eyewitness identification (e.g., Clark et al., 2015).

## Conclusion and implications

It is unlikely that a large number of police departments construct highly biased lineups, as most report that they select fillers by matching to the suspect (Police Executive Research Forum, 2013). Therefore, we argue that eyewitness researchers, rather than comparing very biased with fair lineups, should focus on varying levels of reasonably fair lineups that are more like those used by police. Moreover, we acknowledge that it is not always possible to follow a strict match to description procedure. When the description of a perpetrator is very vague, or when there is a significant mismatch between the description and suspect’s appearance, matching to the suspect can be acceptable, or some combination of the two procedures (see Wells et al., 1998). However, only about 10% of police in the United States select fillers according to the match to description method recommended by the NIJ (Police Executive Research Forum, 2013; Technical Working Group for Eyewitness Evidence, 1999). This is problematic if additional research supports our finding that suspect-matched lineups reduce empirical discriminability.

However, CAC analysis revealed a strong confidence-accuracy relationship regardless of filler selection method, in agreement with recent research on other variables relevant to eyewitness ID (e.g., Semmler, Dunn, Mickes, & Wixted, 2018; Wixted & Wells, 2017). Therefore, although the ROC results indicate that policy makers should recommend that fillers be selected based on match to (a sufficiently detailed) description, the CAC results indicate that judges and juries should not be concerned with which method was utilized in a given case. If an eyewitness provides immediate high confidence in a suspect ID, this carries weight in gauging the likely guilt of the suspect.

## Additional file

Additional file 1: Supplemental material: pilot experiments. (DOCX 36 kb)

## Data Availability

The datasets from these experiments are available from the first author on reasonable request.

## Footnotes

1 We note that there is still some debate in the literature regarding the applicability of ROC analysis to lineup data, with some opposed (e.g., Lampinen, 2016; Smith, Wells, Smalarz, & Lampinen, 2018; Wells, Smalarz, & Smith, 2015), but many in favor (e.g., Gronlund et al., 2012; Gronlund, Wixted & Mickes, 2014; National Research Council, 2014; Rotello & Chen, 2016; Wixted & Mickes, 2012, 2018)

2 We initially conducted three pilot experiments to test our FACES stimuli. See Additional file 1 for information on these experiments.

3 We will refer to the *perpetrator* as the *target* in the results, in order to be consistent with terminology (e.g., target-present and target-absent lineups) from our initial experiments.

4 Most eyewitness researchers do not go to these lengths when creating lineups, but we needed to follow these steps to carefully establish well-operationalized suspect-matched versus description-matched lineups. Prior research following similar steps to create fair lineups has also started with a modal description of the perpetrator, but based on a much smaller group of participants (e.g., *N* = 5; e.g., Carlson, Dias, Weatherford, & Carlson, 2017). We had 10 times as many participants (54) provide descriptions because the resulting modal description was so critical to the purpose of our final experiment, and we therefore wanted it to have a stronger foundation empirically. Later we had only 28 participants choose from each of our lineups the person who best matched the modal description, but this has been shown to be a roughly sufficient number in the literature (e.g., Carlson et al., 2017, based on their Tredoux’s *E’* calculations on 30 participants).

5 In order to ensure that we had a sufficient number of participants for similarity ratings, we had a sample size somewhat larger than another eyewitness ID study featuring pairwise similarity ratings (*N* = 34; Charman, Wells, & Joy, 2011).

6 This specificity is based on the maximum false alarm rate for the most conservative curve (i.e., the shortest curve) so that no extrapolation is required. We repeated all analyses with specificities based on the most liberal curves so that all data from all conditions could be included. The pattern of results in Table 6 remained the same, and overall, suspect-matched lineups (pAUC = .061 [.050–.072]) still had lower discriminability than description-matched lineups (pAUC = .085 [.076–.094]), *D* = 3.30, *p* < .001.

7 With Bonferroni-corrected alpha of .006, one of these four comparisons (Description Match 4 vs. Suspect Match 2; see Table 6) is marginally significant, with *p* = .01. When setting specificity based on the most liberal rather than most conservative condition’s maximum false alarm rate, this difference is significant at *p* = .001.
