# Supplementary material for: Lineup fairness: propitious heterogeneity and the diagnostic feature-detection hypothesis
Source: Cogn Res Princ Implic. 2019 Jun 13;4:20. doi: 10.1186/s41235-019-0172-5 (PMC6565783; doi:10.1186/s41235-019-0172-5)
Supplement: Supplementary file 1 — Supplemental material: pilot experiments. (DOCX 36 kb) [file 41235_2019_172_MOESM1_ESM.docx]

**Supplemental Material: Pilot Experiments**

**Method**

**Participants**

Students from the Texas A&M University – Commerce psychology department subject pool were participants in all three pilot experiments (*N*’s = 44, 46, and 28, respectively). We ran fewer participants in the third pilot compared to the first two because of a simpler design, as described below.

**Materials**

We utilized the FACES 4.0 software (IQ Biometrix, 2003) to create our stimuli (see Figure 1 in the main text for examples). No face had any hair or other distinguishing external characteristics; all shared the same external features as seen in Figure 1. The only features that varied were the eyes, nose, and/or mouth. In Pilot Experiment 1, only one of these features varied in a given lineup. In Pilot Experiment 2, two of these three features varied in a given lineup, thereby providing participants with more featural information on which to base their ID decision (again, for both TP and TA lineups). Lastly, all three features varied in every lineup of Pilot Experiment 3. Each target was randomly assigned to a position during creation of the TP lineups, and there was no designated innocent suspect in TA lineups.

**Design and Procedure**

All three pilot experiments involved within-subjects designs: (a) Experiment 1: 3 (eyes, nose, or mouth varying in each lineup) x 2 (TP or TA lineup); (b) Experiment 2: 3 (eyes and nose, eyes and mouth, or nose and mouth varying in each lineup) x 2 (TP or TA lineup); (c) Experiment 3 had just one factor: TP or TA lineup, as all three facial features varied in every lineup. No face or feature ever repeated across lineups or experiments, except the external characteristics (e.g. face shape, ears), which remained constant for all faces in all experiments (see Figure 1).

Procedurally, for all three pilot experiments participants took part in a face recognition paradigm with 18 blocks, with each containing an encoding phase, a distractor task, and then a lineup. During each encoding phase, a series of 20 real faces were presented sequentially for 0.5 s each, and a computer-generated face (the target) was presented in quasi-random position toward the middle of this presentation (for 1 s). Participants were instructed to study all of these faces, as they would be tested on them later in that block. The distractor task in each block was a yes/no recognition test for the real faces (10 old and 10 new), and we did not analyze these data. The final part of each block was a simultaneous lineup of six FACES presented in a 2x3 array, and participants were instructed to identify the target presented earlier in that block, which may or may not be present (half of the 18 blocks were TP, and half were TA, in randomized order). They could choose one of the six lineup members or reject the lineup. After their decision, they entered their confidence on an 11-point scale (0-100% in 10% increments), and then the next block automatically began.

**Results**

The most direct tests of our hypotheses required collapsing over the different features that we manipulated. In other words, for Pilot E1 we collapsed across manipulations of eyes, nose, and mouth to have one set of data involving a single feature varying in each lineup. Similarly, for Pilot E2, we collapsed across eyes-nose, eyes-mouth, and nose-mouth lineups to create a single set of data involving two features varying. All three features varied in all lineups in Pilot E3, so no collapsing was necessary. We applied the Bonferroni adjustment for multiple comparisons, setting α = .05/3 = .017. We will first describe the results of ROC analysis, followed by logistic regression and chi-square analyses to assess differences in the number of correct IDs from TP lineups separately from the number of false IDs from TA lineups (Gronlund & Neuschatz, 2014). See Table S1 for all lineup decisions.

| Table S1. |  |  |  |  |  |
| --- | --- | --- | --- | --- | --- |
|  |  |  |  |  |  |
| Number of identifications and rejections from pilot experiments | | | |  |  |
|  |  |  |  |  |  |
| **Condition** | **Target-Present Lineups** | | | **Target-Absent Lineups** | |
|  | **Correct ID Rate** | **Filler ID Rate** | **Rejection Rate** | **Filler ID Rate** | **Rejection Rate** |
| One Feature Varies | 0.68 | 0.18 | 0.14 | 0.66 | 0.34 |
| Two Features Vary | 0.73 | 0.14 | 0.13 | 0.46 | 0.54 |
| Three Features Vary | 0.77 | 0.08 | 0.15 | 0.34 | 0.66 |
| *Note: ID = Identification* | |  |  |  |  |

**ROC Analysis**

As seen in Figure S1, our prediction derived from the DFD hypothesis (Wixted & Mickes, 2014) was supported, such that empirical discriminability increased as the number of diagnostic features increased. The pAUCs were as follows: (a) 1 feature varied, pAUC = .056 [95% CIs: .047 - .067]; (b) 2 features varied, pAUC = .070 [.061 - .079]; and (c) 3 features varied, pAUC = .084 [.074 - .093]. Discriminability increased marginally from one to two features, *D* = 2.04, *p* = .04, and marginally from two to three features, *D* = 2.06, *p* = .04. Finally, three features yielded significantly greater discriminability than just one feature, *D* = 3.87, *p* < .001.


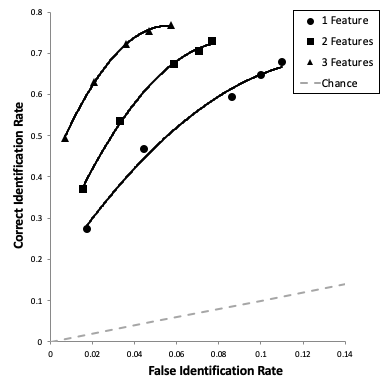


*Figure S1.* ROC data from the three Pilot Experiments. The curves drawn through the empirical data points are not based on model fits, but rather are simple trendlines drawn in Excel. The correct ID rate on the y-axis is the proportion of targets chosen from the total number of target-present lineups in a given condition. The false ID rate on the x-axis is the proportion of all filler identifications from the total number of target-absent lineups in a given condition (as we had no designated innocent suspects), divided by the nominal lineup size (six) in order to provide an estimated innocent suspect ID rate.

**Separate Analyses of TP and TA Lineups**

The full model comparing correct IDs across the three experiments was marginally significant, Wald (2) = 5.92, *p* = .052. There were marginally more correct IDs when two features varied compared to one feature, χ2 (1, *N* = 810) = 2.45, *p* = .068, ϕ = .06, and significantly more correct IDs when three features varied compared to one feature, χ2 (1, *N* = 620) = 5.47, *p* = .012, ϕ = .09. There was no difference in correct IDs when two versus three features varied, χ2 (1, *N* = 638) = 1.12, *p* = .168, ϕ = .04.

Turning to the false IDs, these were simply the total number of IDs from TA lineups. Unlike the marginally significant model for correct IDs, the full model for false IDs was significant, Wald (2) = 62.97, *p* < .001. Specifically, the number of false IDs was inversely proportional to the number of features that varied in each lineup. There were more false IDs when only one feature varied, compared to two features, χ2 (1, *N* = 810) = 32.93, *p* < .001, ϕ = .20, and compared to three features, χ2 (1, *N* = 620) = 58.33, *p* < .001, ϕ = .31. There were also more false IDs when two features varied compared to three features, χ2 (1, *N* = 638) = 8.25, *p* = .003, ϕ = .11.

**Discussion**

In support of other research investigating lineups of high filler similarity (e.g., Fitzgerald et al., 2015), these pilot experiments indicate that lineups containing very similar fillers could be problematic, as they tended to lower ID accuracy (see also simulations by Clark et al., 2013). We went a step beyond this research to show with ROC analysis that empirical discriminability declines at the upper levels of filler similarity. Allowing more features to vary among lineup members generally increased accuracy. These preliminary findings support the principle of propitious heterogeneity (e.g., Wells et al., 1993) and the DFD hypothesis (Wixted & Mickes, 2014).
